# A mouse model of fenugreek allergy

**DOI:** 10.1186/2045-7022-1-S1-P48

**Published:** 2011-08-12

**Authors:** Nina E Vinje, Ellen Namork, Martinus Lovik

**Affiliations:** 1 Norwegian Institute of Public Health, Dept. of Environmental Immunology, Oslo, Norway

## Background

Fenugreek (*Trigonella foenum-graecum L.*) is widely used in Indian-style spiced foods. Some cases of allergic reactions to fenugreek have been reported, and a Norwegian study found cross-reactivity of fenugreek in peanut allergic individuals. Thus, we wanted to develop a mouse model of fenugreek allergy.

## Methods

Female C3H/HeJ mice were gavaged at days 0, 1, 2, 7, and 21 with fenugreek in doses ranging from 0.1 mg to 10 mg. Cholera toxin (CT) was used as an adjuvant (10 µg/mouse). The mice were euthanized on day 28, and sera were analysed for fenugreek-specific IgE using the heterologous passive cutaneous anaphylaxis assay. Based on this dose-response experiment, twenty four mice were gavaged with the optimal dose of fenugreek (4.2 mg) and CT at days 0, 1, 2, 7, 21 and 28. Eight mice were gavaged with CT only and 8 mice were not treated. At day 35 all mice received a large dose of fenugreek (25.0 mg) and clinical reactions were observed.

## Results

We found the optimal dose that would elicit the strongest IgE response to be 4.24 mg fenugreek. Provocation with fenugreek in immunised animals resulted in clinical reactions like scratching and rubbing around the nose and head, puffiness around the eyes and mouth as well as laboured respiration.

## Conclusion

We have established a mouse model of fenugreek allergy to be used in further studies of primary fenugreek allergy and in cross-reactions with other legumes.

